# Identification of ESBL‐Producing Enterobacterales From Vegetable Plants: Preliminary Findings From a Small Cross‐Sectional Study in a Rural Area of Madagascar

**DOI:** 10.1111/1758-2229.70130

**Published:** 2025-06-17

**Authors:** Adrien Rieux, Mamitina Alain Noah Rabenandrasana

**Affiliations:** ^1^ CIRAD, UMR PVBMT, F‐97410 St Pierre La Réunion France; ^2^ Experimental Bacteriology Unit Institut Pasteur de Madagascar Antananarivo Madagascar

## Abstract

Extended‐spectrum beta‐lactamases (ESBL)‐producing enterobacterales are considered a key indicator for antimicrobial resistance (AMR) epidemiological surveillance in animal, human, and environment compartments. In this study, we aim to investigate the presence and genetic diversity of ESBL‐producing enterobacterales on vegetable plants. We isolated beta‐lactam resistant enterobacterales from several vegetable plants and sequenced their whole genome. Utilising standard genomic and phylogenetic methods, we sought to (i) characterise the resistance genes and plasmid content of the plant‐isolated strains, (ii) investigate their genetic structure, and (iii) determine their relationships with strains from other reservoirs. Among the 22 strains collected from vegetable plants, 6 showed resistance to beta‐lactam antibiotics, with 5 of them identified as ESBL producers. Our results indicated the presence of multidrug‐resistant (MDR) strains containing multiple antibiotic resistance genes (ARGs). Importantly, no host‐specific lineages were identified among the plant‐isolated ESBL‐producing *E. coli* (ESBL‐Ec). Instead, these strains exhibited genetic and epidemiological connections with strains isolated from animals, humans, and the environment, suggesting transfer of ESBL‐Ec between plants and other sources in rural Madagascar. These preliminary findings suggest that vegetable plants are contaminated as a result of human activities, posing a potential risk of human and animal exposure to antibiotic‐resistant bacteria and genes.

## Graphical abstract

In this pilot study, multidrug‐resistant ESBL‐producing enterobacterales were detected on several vegetable plants in rural Madagascar. Genomic analysis uncovered epidemiological connections between plant‐associated strains and those originating from humans, animals, and environmental sources, underscoring the potential for cross‐reservoir transmission of antimicrobial resistance.

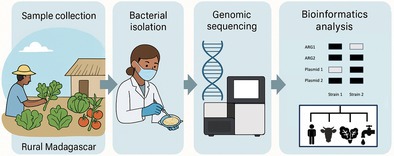

## 1 Introduction

Extended‐spectrum beta‐lactamase (ESBL)‐producing Gram‐negative bacteria are a leading cause of human and animal infection, being categorised as critically important pathogens by the World Health Organization (Tacconelli et al. 2018). In this context, antimicrobial resistance (AMR) is recognised as a major One Health challenge because of the rapid emergence and dissemination of resistant bacteria and genes among humans, animals and the environment (McEwen and Collignon 2018). Aside from assessing AMR prevalence, there is an increasing need to better understand behaviours, customs and practices that drive the evolution and transmission of resistance, both globally and in low‐resource settings in which the AMR threat is of particular concern (Rousham et al. 2018). In this context, the use of antibiotics in agriculture has historically been described as a major contributortof AMR in humans due to a large variety of potential dissemination pathways, with most of them involving the influence of livestock (Thanner et al. 2016). The role of livestock in the emergence of AMR was shown to be multifaceted, involving both direct transmission to humans and environmental contamination (Bava et al. 2024). In contrast, little is known about the existence and prevalence of antimicrobial resistant bacteria and resistance genes (ARB & ARG, respectively) within the cultivated plant reservoir, despite the fact that such food is often consumed raw, hence increasing the risk of transmission to humans (Hölzel et al. 2018). Herein, we aimed to characterise the prevalence and genetic diversity of beta‐lactam resistant enterobacteriaceae isolated from vegetable plants in a rural area of Madagascar. To assess the genetic and epidemiological relationships between strains isolated from plants and other reservoirs, we integrated data from a cross‐sectional population‐based recent study performed within the same area on animals, humans and the environment (Gay et al. 2023).

## 2 Experimental Procedures

### 2.1 Sampling, Bacterial Culture and Sequencing

In September 2018, 22 vegetable plants (spinach, lettuce, tomato and cabbage) were sampled within 6 backyard gardens in Andoharanofotsy, Madagascar (Figure S1). Sampling was performed using sterile equipment. Plant leaves were transported back to the laboratory in individual envelopes before being finely cut and soaked in LB broth for 24 h at 35°C ± 2°C with continuous shaking. 100 μL of the enriched suspension was directly streaked onto selective chromogenic agar plates (CHROMagar ESBL; CHROMagar, Paris, France) and incubated overnight at 35°C ± 2°C under aerobic conditions. All presumptive ESBL‐producer morphotypes were subcultured individually on LB agar plates.

In vitro antimicrobial susceptibility testing was performed on one isolate according to the standard disc methods described in the 2024 ‘Comité de l'Antibiogramme de la Société Française de Microbiologie’ (CASFM)‐EUCAST guidelines (Cattoir 2024). The presence of ESBL enzymes was confirmed by synergy of cefotaxime, ceftazidime and cefepime with amoxicillin/clavulanate or ticarcillin/clavulanate.

One bacterial isolate per sample was randomly selected for genetic analysis. DNA extraction was performed using the Cador Pathogen Extraction Kit (INDICAL Bioscience) from 5 mL of liquid cultures grown overnight at 37°C in LB broth medium. Library preparation was performed using the Nextera XT DNA Library Preparation Kit (Illumina, San Diego, CA, USA) and sequencing was performed on a NextSeq 500 platform (Illumina) using 2 × 150 bp runs.

### 2.2 Core Genome Analyses

Raw reads were trimmed with Trimmomatic (Bolger et al. 2014) to remove adapters and low‐quality sequences before being *de novo* assembled using the Unicycler pipeline (Wick et al. 2017) (default parameters). Taxonomic assignment was performed on assembled bacterial genomes both by computing ANI using FastANI (Jain et al. 2018) and by running Kraken on the microbial reference database (Wood and Salzberg 2014). In order to investigate the phylogenetic structure of the plant‐isolated ESBL genomes within a global One‐health framework, we aligned the bacterial genomes generated within the course of this study with a public dataset of 510 ESBL strains also isolated in 2018 from human, animal and water in the same rural area of Madagascar (Gay et al. 2023). Genomes were aligned using parsnp (Treangen et al. 2014) and regions acquired via horizontal gene transfers were detected using Gubbins (Croucher et al. 2015). From the recombination‐free SNP alignment, a maximum likelihood phylogeny was constructed using RAxML (Stamatakis 2014) with a rapid bootstrap analysis, general time‐reversible model of evolution with a four rate categories γ distribution (GTRGAMMA) and 1000 iterations. Transmission clusters were inferred from the reconstructed phylogeny using the dedicated Phydelity tool (Han et al. 2019).

### 2.3 Genotyping, Mobilome and Resistome Analysis

Sequence types and phylogroups were estimated *in silico* using staram (Bharat et al. 2022) and ClermonTyping (Beghain et al. 2018) softwares, respectively. Staramr was also used to scan bacterial genome contigs against the ResFinder, PointFinder, and PlasmidFinder databases to detect the presence of plasmids and antimicrobial resistance genes. RFPlasmid (van der Graaf‐van Bloois et al. 2021) was used to predict whether the detected ARGs were located on plasmids or chromosomes.

## 3 Results

### 3.1 Prevalence of Beta‐Lactam Resistant and ESBL Producing Enterobacterales From Vegetable Plants

**FIGURE 1:**
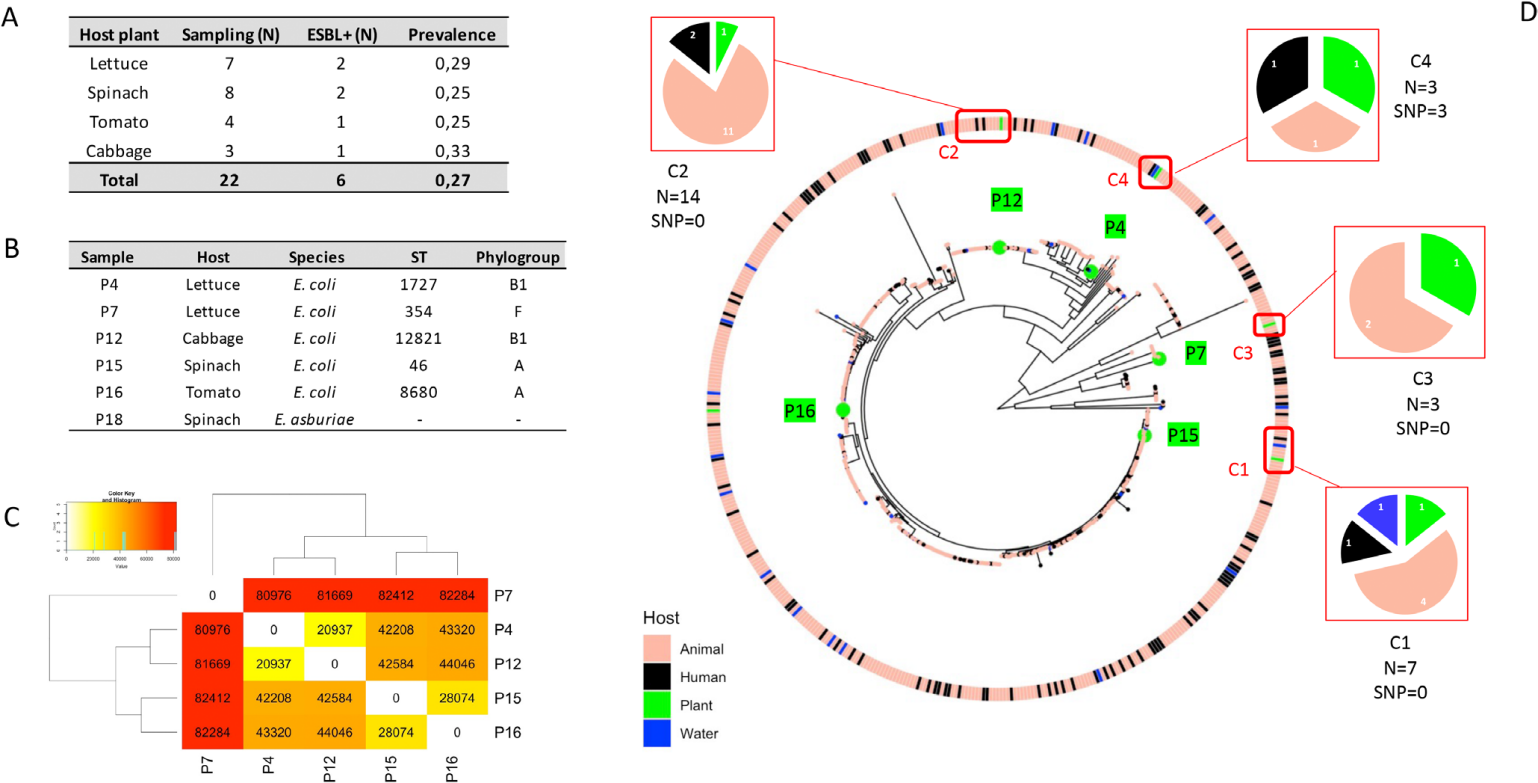
(A) Sampled plants and prevalence of isolated beta‐lactam resistant enterobacterales. (B) Taxonomic assignment of isolated enterobacterales. (C) Pairwise SNP number between the five ESBL‐Ec. (D) Phylogenetic maximum likelihood tree of 515 ESBL‐Ec genomes built from 329,817 core and non‐recombinant SNPs. Hosts for each isolate (human, animal, plant, water) are depicted using different colours, with plant isolates highlighted in green. Transmission clusters and their composition are indicated with red frames and pie charts, respectively. The number of isolates in each cluster (N) and their average SNP count (SNP) are shown below.

We identified beta‐lactam resistant enterobacterales from 6 out of 22 collected plants (average prevalence of 27%, Figure 1A). Resistant isolates were isolated from 5 out of the 6 sampled backyard gardens and originated from lettuce (*n* = 2), spinach (*n* = 2), cabbage (*n* = 1) and tomato (*n* = 1) (Figure 1B).

In vitro antimicrobial susceptibility testing yielded variable results according to the strains and antibiotics tested (Figure S2). All 6 strains were sensitive to Ertapenem & Imipenem, resistant to Amoxicillin, Ceftazidim & Cefotaxim and showed variable sensitivity patterns towards the other tested antibiotics. Five strains (P4, P7, P12, P15 and P16) passed the synergy test and were hence considered as ESBL producers. Strain P18, although resistant to cefotaxime, ceftazidime, ticarcillin/clavulanate & amoxicillin/clavulanate did not produce any ESBL.

### 3.2 Bacterial Sequencing

Sequencing generated around 8 million paired‐end reads with a majority (99.90%–99.96%) of bases scoring Q30 and above. *Denovo* assembly statistics are given in Table S1.

### 3.3 Taxonomic Assignment, Mobilome and Resistome

Out of the 6 beta‐lactam resistant enterobacterales, 5 were assigned to *Escherichia coli* and 1 to *Enterobacter asburiae* species. The five *E. coli* , all ESBL producers, were assigned to three different phylogroups (A; *n* = 2, B1; *n* = 2 and F; *n* = 1) and 5 STs (1727, 354, 12,821, 46 & 880), respectively.

**FIGURE 2:**
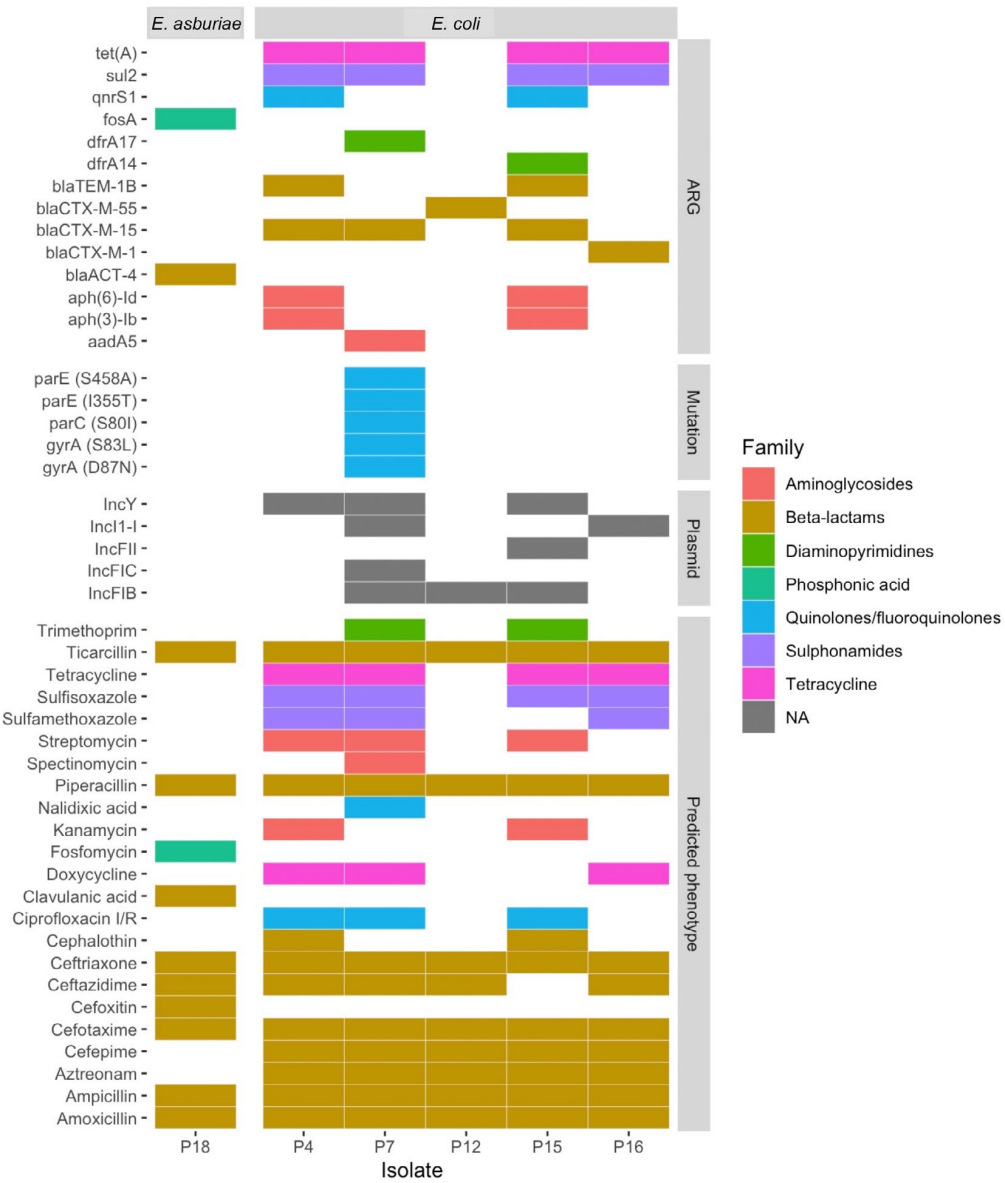
Distribution of AMR genotypes and predicted resistance phenotypes among the six plant‐isolated beta‐lactam resistant enterobacterales strains. Filled boxes indicate the presence of ARGs, point mutations, plasmids, or a predicted antibiotic‐resistant phenotype, with colours corresponding to different antibiotic families.

A total of 14 different ARGs, 5 point mutations and 5 plasmids were identified among the six plant beta‐lactam resistant enterobacterales (Figure 2 and Table S2). In total, the six isolated strains harboured ARGs against seven different antibiotic families. The five ESBL‐Ec strains were predicted to be resistant to at least three antibiotic families and hence considered as multiple drug resistant (MDR). All six strains were predicted to be resistant to several antibiotics from the Beta‐lactams family. When comparing resistance patterns predicted *in silico* with those determined in vitro, we observed consistent results for 94% (74/78) of the strains/antibiotics tested (Figure S3).

The most frequently detected ESBL‐gene was blaCTX‐M‐15, and a total of 5 different plasmids (IncY, IncFIB, IncFIC, IncFII and IncI1‐I) were shown to segregate within the sampled strains. Detected ARGs were predicted to be carried either by chromosomes or plasmids (Table S2) except for *bla*_CTX‐M‐15_, which was detected both on a chromosome (strain P7) and a plasmid (strain P4). Most detected ARGs displayed very high nucleotide identity rates with reference genes (Table S2).

### 3.4 Genomic Diversity and Phylogenetic Structure

As illustrated by their assignment to unique STs, the five ESBL‐Ec strains isolated from plants displayed high levels of genomic diversity with a mean pairwise SNP number = 54,851 (Figure 1C). In order to decipher their genetic relationships with strains isolated from other reservoirs, we built a phylogenetic tree using a dataset composed of previously published ESBL‐Ec genomic resources (Gay et al. 2023) (Total of 329,817 SNPs in between 515 ESBL‐Ec strains). Interestingly, plant ESBL‐Ec strains did not cluster together within the phylogenetic tree but were instead intermixed within other human/animal/environment‐isolated strains (Figure 1D). Furthermore, among the five plant ESBL‐Ec strains, four were assigned to transmission clusters with other strains originating from other compartments (Figure 1D and Table S3). Strain P16, isolated from tomato leaf, was not assigned to any transmission cluster.

## 4 Discussion

In this study, we report for the first time the presence of ESBL‐producing enterobacterales from vegetable plants sampled in Madagascar. In this regard, the occurrence of ESBL producers in various crops and fresh vegetables has previously been described in other countries (Hölzel et al. 2018; Lopes et al. 2021; Reuland et al. 2014; Zeynudin et al. 2025; Tresch et al. 2024; Zurfluh et al. 2015). Interestingly, members of the enterobacterales order have previously been shown to exhibit both an endophytic (i.e., colonise internal tissues) and epiphytic (i.e., live and multiply on the outside of aerial surfaces) lifestyle on vegetable plants (Lopes et al. 2021). Unfortunately, the methodology used in this study did not allow for a distinction between the two lifestyle modes. Among the five *E. coli* strains recovered, isolate P15 sampled from spinach was assigned to ST46, which encompasses pathogenic strains responsible for septicaemia, diarrhoea, meningitis, and urinary tract infections (Vignaroli et al. 2016). In addition, P18, also isolated from spinach, was assigned to *E. asburiae* , a bacterial species previously shown to be associated with humans and animals while also causing disease in plants such as rice and radish (Xue et al. 2021; Wang et al. 2023). One limitation of this study is that only a single bacterial isolate was analysed per sample, which, while practical for a preliminary investigation, may introduce selection bias and potentially overlook the presence of other co‐existing resistant strains within the same sample.

Determinants of antimicrobial resistance and plasmids were *in silico* detected from the genomic data. Among the detected genes providing resistance to beta‐lactams, b*la*_CTX‐M‐15_ and b*la*_CTX‐M‐55_ are both members of the CTX‐M‐1 group, considered as one of the most predominant enzymes in the widespread dissemination of ESBLs (Yang et al. 2023). Interestingly, those ESBL genes have previously been detected within different compartments in Madagascar (Gay et al. 2023; Milenkov et al. 2021), highlighting their relevance to One Health concerns. In this context, a recent study revealed frequent and multiple transmission events between ESBL‐Ec strains isolated from humans, animals, and the environment (drinking water) in a suburban rural area of Antananarivo, Madagascar (Gay et al. 2023). Through a dedicated phylogenetic analysis, we demonstrated that the newly isolated ESBL‐Ec strains from vegetable plants were interspersed within the genomic diversity of strains from the other compartments, as illustrated by the inference of shared transmission clusters. The lack of genomic diversity within the detected clusters supports a likely recent epidemiological connection between strains from vegetable plants and other reservoirs. Although the origins of ESBL‐producing enterobacterales found in this study on vegetable plants were not investigated, they could originate from human (as sewage), animal (manure), and/or environmental (such as contaminated soil and irrigation water) sources that come into contact with crops (Beuchat 2002; Araújo et al. 2017). Therefore, our pilot results suggest that the consumption of raw vegetables should be seen as a public health problem and a potential risk of human exposure to antibiotic‐resistant bacteria and/or their resistance genes. Future studies performed on a higher number of samples are required to decipher more accurately the drivers and pathways of ARB & ARG transmission between plants and other compartments, in order to gather data for risk assessment.

## Author Contributions

A.R. designed the study and performed field sampling. M.A.N.R. performed lab work and assisted with the sequencing work. A.R. and M.A.N.R. both performed genomic analyses and wrote the manuscript.

## Conflicts of Interest

The authors declare no conflicts of interest.

## Supporting information


**Figure S1.** Sampling sites in Andoharanofotsy, Madagascar.
**Figure S2.** In vitro antimicrobial susceptibility results.
**Figure S3.** Comparison between measured and predicted resistant phenotypes.
**Table S1.** Genomic data generated for the 6 plant‐isolated enterobacterales.
**Table S2.** Antibiotic resistant genes and plasmids *in silico* identified within the plant‐isolated enterobacterales.
**Table S3.** Composition of ESBL‐Ec transmission clusters (estimated with Phidelity) involving plant‐isolated strains.

## Data Availability

The data that support the findings of this study are openly available in ncbi at https://www.ncbi.nlm.nih.gov/, reference number PRJNA1147432.
