# Supplementary material for: Identification of ESBL‐Producing Enterobacterales From Vegetable Plants: Preliminary Findings From a Small Cross‐Sectional Study in a Rural Area of Madagascar
Source: Environ Microbiol Rep. 2025 Jun 17;17(3):e70130. doi: 10.1111/1758-2229.70130 (PMC12173957; doi:10.1111/1758-2229.70130)
Supplement: Supplementary file 1 — Figure S1. Sampling sites in Andoharanofotsy, Madagascar. Figure S2. In vitro antimicrobial susceptibility results. Figure S3. Comparison between measured and predicted resistant phenotypes. Table S1. Genomic data generated for the 6 plant‐isolated enterobacterales. Table S2. Antibiotic resistant genes and plasmids in silico identified within the plant‐isolated enterobacterales. Table S3. Composition of ESBL‐Ec transmission clusters (estimated with Phidelity) involving plant‐isolated strains. [file EMI4-17-e70130-s001.pdf]

Sample	Site	GPS coord	Vegetable plant	ESBL
P1	S1	S 18,99408 E 047,54015	spinach	Negative
P2	S1	S 18,99408 E 047,54015	lettuce	Negative
P3	S1	S 18,99408 E 047,54015	lettuce	Negative
P4	S1	S 18,99408 E 047,54015	lettuce	Positive
P5	S2	S 18,99916 E 047,53862	spinach	Negative
P6	S2	S 18,99916 E 047,53862	tomato	Negative
P7	S2	S 18,99916 E 047,53862	lettuce	Positive
P8	S3	S 18,99064 E 047,53586	spinach	Negative
P9	S3	S 18,99064 E 047,53586	cabbage	Negative
P10	S3	S 18,99064 E 047,53586	lettuce	Negative
P11	S3	S 18,99064 E 047,53586	lettuce	Negative
P12	S3	S 18,99064 E 047,53586	cabbage	Positive
P13	S4	S 18,97230 E 047,42816	cabbage	Negative
P14	S4	S 18,97230 E 047,42816	spinach	Negative
P15	S4	S 18,97230 E 047,42816	spinach	Positive
P16	S4	S 18,97230 E 047,42816	tomato	Positive
P17	S5	S 18,97200 E 047,52409	lettuce	Negative
P18	S5	S 18,97200 E 047,52409	spinach	Positive
P19	S6	S 18,99850 E 047,54042	spinach	Negative
P20	S6	S 18,99850 E 047,54042	spinach	Negative
P21	S6	S 18,99850 E 047,54042	tomato	Negative
P22	S6	S 18,99850 E 047,54042	tomato	Negative

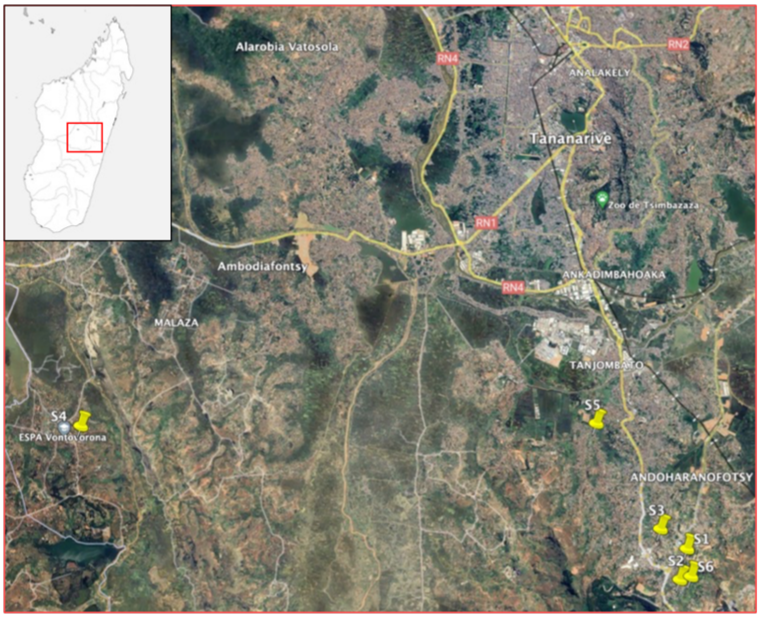

Fig S1: Sampling sites in Andoharanofotsy, Madagascar

ID	P4	P7	P12	P15	P16	P18
Amoxicillin	6	6	6	6	6	6
Amoxicillin-clavulanate	20	22	21	20	22	6
Aztreonam	18	19	16	19	18	30
Cefepim	16	18	18	18	19	40
Cefotaxim	6	6	6	9	6	6
Cefoxitin	21	21	26	27	22	6
Ceftazidim	18	16	15	18	17	14
Ciprofloxacin	29	10	31	31	32	46
Ertapenem	25	26	26	25	29	28
Imipenem	30	30	32	29	31	30
Nalidixic Acid	25	11	23	23	24	38
Ticarcillin-clavulanate	23	24	25	24	24	6
Trimethoprim-Sulfamethoxazole	21	10	23	11	22	30
Synergy_test_ESBL	Positive	Positive	Positive	Positive	Positive	Negative

(red)	*In vitro* measured resistant phenotype
(green)	*In vitro* measured sensitive phenotype

Values indicate diameters of bacterial growth inhibition measured on agar plates (in mm)

Fig S2: *In vitro* antimicrobial susceptibility results.

ID	P4	P7	P12	P15	P16	P18
Amoxicillin	R	R	R	R	R	R
Amoxicillin-clavulanate	S	S	S	S	S	R
Aztreonam	R	R	R	R	R	S
Cefepim	R	R	R	R	R	S
Cefotaxim	R	R	R	R	R	R
Cefoxitin	S	S	S	S	S	R
Ceftazidim	R	R	R	S	R	R
Ciprofloxacin	R	R	S	R	S	S
Ertapenem	S	S	S	S	S	S
Imipenem	S	S	S	S	S	S
Nalidixic Acid	S	R	S	S	S	S
Ticarcillin-clavulanate	S	S	S	S	S	R
Trimethoprim-Sulfamethoxazole	S	R	S	S	S	S

	In vitro -measured resistant phenotype
	In vitro -measured sensitive phenotype
R	In siico-predicted resistant phenotype
S	In siico-predicted sensitive phenotype

Discrepancy between measured & predicted patterns

Fig S3: Comparison between measured and predicted resistant phenotypes

Sample	P4	P7	P12	P15	P16	P18
Vegetable plant	Salad	Salad	Cabbage	Spinach	Tomato	Spinach
Scientific name	*Lactuca sativa*	*Lactuca sativa*	*Brassica oleracea*	*Spinacia oleracea*	*Solanum lycopersicum*	*Spinacia oleracea*
Taxonomic assignment	*Esherichia coli*	*Esherichia coli*	*Esherichia coli*	*Esherichia coli*	*Esherichia coli*	*Enterobacter cloacae*
ST	1727	354	12821	46	8680	-
Phylogroup	B1	F	B1	A	A	-
Genome size (Gb)	4,64	5,09	4,76	4,63	4,48	4,68
Number of contigs	107	250	171	174	191	42
N50 value	72520	49481	51290	55944	42774	171452
Bioproject	PRJNA1147432					
BioSample	SAMN43154107	SAMN43154108	SAMN43154109	SAMN43154110	SAMN43154111	SAMN43154112
SRA (raw reads)	SRR30230049	SRR30230048	SRR30230047	SRR30230046	SRR30230045	SRR30230044
Assembled genome	JBGMDQ000000000	JBGMDP000000000	JBGMDO000000000	JBGMDN000000000	JBGMDM000000000	JBGMDL000000000

Table S1: Genomic data generated for the 6 plant-isolated enterobacterales

Isolate ID	Data	Data Type	Predicted Phenotype	Genetic support (probability)	%Identity	%Overlap	HSP Length/Total Length	Contig	Start	End	Accession
**P12**	IncFIB	Plasmid		Plasmid (0.71)	100	100	560/560	149	464	1023	CU638872
**P12**	blaCTX-M-55	Resistance	Amoxicillin, Ampicillin, Aztreonam, Cefepime, Cefotaxime, Ceftriaxone, Ceftazidime, Piperacillin, Ticarcillin	Chromosome (1)	100	100	876/876	152	1144	2019	DQ810789
**P15**	IncFIB(AP001918)	Plasmid		Plasmid (0.87)	99,12	100	682/682	114	1835	2516	AP001918
**P15**	IncFII	Plasmid		Plasmid (0.99)	96,56	100	261/261	42	1662	1923	AY458016
**P15**	IncY	Plasmid		Plasmid (0.90)	98,82	100	765/765	50	27922	28686	K02380
**P15**	aph(3'')-Ib	Resistance	Streptomycin	Plasmid (0.95)	100	100	804/804	36	35754	34951	AF321551
**P15**	aph(6)-Id	Resistance	Streptomycin, kanamycin	Plasmid (0.95)	100	100	837/837	36	34951	34115	M28829
**P15**	blaCTX-M-15	Resistance	Amoxicillin, Ampicillin, Aztreonam, Cefepime, Cefotaxime, Ceftazidime, Ceftriaxone, Piperacillin, Ticarcillin	Plasmid (0.95)	100	100	876/876	36	29712	28837	AY044436
**P15**	blaTEM-1B	Resistance	Amoxicillin, Ampicillin, Cephalothin, Piperacillin, Ticarcillin	Plasmid (0.95)	100	100	861/861	36	32534	33394	AY458016
**P15**	dfrA14	Resistance	Trimethoprim	Plasmid (0.72)	100	100	474/474	142	1162	1635	KF921535
**P15**	qnrS1	Resistance	Ciprofloxacin	Plasmid (0.95)	100	100	657/657	36	24196	23540	AB187515
**P15**	sul2	Resistance	Sulfamethoxazole, Sulfisoxazole	Plasmid (0.95)	100	100	816/816	36	36630	35815	AY034138
**P15**	tet(A)	Resistance	Doxycycline, Tetracycline	Plasmid (0.91)	100	100	1200/1200	124	1054	2253	AJ517790
**P16**	IncI1-I(Alpha)	Plasmid		Plasmid (0.93)	99,3	100	142/142	63	5034	5175	AP005147
**P16**	blaCTX-M-1	Resistance	Amoxicillin, Ampicillin, Aztreonam, Cefepime, Cefotaxime, Ceftazidime, Ceftriaxone, Piperacillin, Ticarcillin	Plasmid (0.99)	100	100	876/876	8	13545	14420	DQ915955
**P16**	sul2	Resistance	Sulfamethoxazole, Sulfisoxazole	Plasmid (0.93)	100	100	816/816	63	16389	17204	AY034138
**P16**	tet(A)	Resistance	Doxycycline, Tetracycline	Plasmid (0.93)	100	100	1200/1200	63	12731	11532	AJ517790
**P18**	blaACT-4	Resistance	Amoxicillin, Amoxicillin+Clavulanic acid, Ampicillin, Ampicillin+Clavulanic acid, Cefotaxime, Ceftriaxone Cefoxitin, Ceftazidime, Piperacillin, Piperacillin+Tazobactam, Ticarcillin, Ticarcillin+Clavulanic acid	Chromosome (0.98)	96,34	100	1146/1146	14	17359	16214	AJ311172
**P18**	fosA	Resistance	Fosfomycin	Plasmid (0.99)	95,3	100	426/426	2	311450	311025	AEXB01000013
**P4**	IncY	Plasmid		Plasmid (0.82)	98,82	100	765/765	60	23140	23904	K02380
**P4**	aph(3'')-Ib	Resistance	Streptomycin	Plasmid (0.95)	100	100	804/804	81	2278	1475	AF321551
**P4**	aph(6)-Id	Resistance	Streptomycin, kanamycin	Plasmid (0.95)	100	100	837/837	81	1475	639	M28829
**P4**	blaCTX-M-15	Resistance	Amoxicillin, Ampicillin, Aztreonam, Cefepime, Cefotaxime, Ceftazidime, Ceftriaxone, Piperacillin, Ticarcillin	Plasmid (0.89)	100	100	876/876	78	6292	5417	AY044436
**P4**	blaTEM-1B	Resistance	Amoxicillin, Ampicillin, Cephalothin, Piperacillin, Ticarcillin	Plasmid (0.89)	100	100	861/861	78	9114	9946	AY458016
**P4**	qnrS1	Resistance	Ciprofloxacin	Plasmid (0.89)	100	100	657/657	78	776	120	AB187515
**P4**	sul2	Resistance	Sulfamethoxazole, Sulfisoxazole	Plasmid (0.95)	100	100	816/816	81	3154	2339	AY034138
**P4**	tet(A)	Resistance	Doxycycline, Tetracycline	Plasmid (0.72)	100	100	1200/1200	87	2848	1649	AJ517790
**P7**	IncFIB(AP001918)	Plasmid		Plasmid (0.73)	98,53	100	682/682	165	2629	1948	AP001918
**P7**	IncFIC(FII)	Plasmid		Plasmid (0.93)	95,79	100	499/499	83	13564	13068	AP001918
**P7**	IncI1-I(Alpha)	Plasmid		Plasmid (0.75)	94,93	97,18	138/142	182	2237	2102	AP005147
**P7**	IncY	Plasmid		Plasmid (0.72)	99,08	100	765/765	106	3159	3923	K02380
**P7**	aadA5	Resistance	Spectinomycin, Streptomycin	Plasmid (0.97)	100	100	789/789	80	926	138	AF137361
**P7**	blaCTX-M-15	Resistance	Amoxicillin, Ampicillin, Aztreonam, Cefepime, Cefotaxime, Ceftazidime, Ceftriaxone, Piperacillin, Ticarcillin	Chromosome (0.91)	100	100	876/876	20	4279	3404	AY044436
**P7**	dfrA17	Resistance	Trimethoprim	Plasmid (0.97)	100	100	474/474	80	1530	1057	FJ460238
**P7**	gyrA (D87N)	Resistance	Nalidixic acid;Nalidixic acid,Ciprofloxacin	Chromosome (1)	97,83	100	2628/2628	53	26230	23603	
**P7**	gyrA (S83L)	Resistance	Nalidixic acid,Ciprofloxacin	Chromosome (1)	97,83	100	2628/2628	53	26230	23603	
**P7**	parC (S80I)	Resistance	Nalidixic acid,Ciprofloxacin	Chromosome (1)	98,05	100	2259/2259	24	21427	23685	
**P7**	parE (I355T)	Resistance	Nalidixic acid,Ciprofloxacin	Chromosome (1)	96,4	100	1893/1893	24	6260	8148	
**P7**	parE (S458A)	Resistance	Nalidixic acid,Ciprofloxacin	Chromosome (1)	96,4	100	1893/1893	24	6260	8148	
**P7**	sul2	Resistance	Sulfamethoxazole, Sulfisoxazole	Plasmid (0.92)	100	100	816/816	148	1444	2259	AY034138
**P7**	tet(A)	Resistance	Doxycycline, Tetracycline	Plasmid (0.97)	100	100	1200/1200	80	8048	9247	AJ517790

Table S2: Resistome and plasmidome of the plant-isolated enterobacterales

Cluster ID	Sample ID	Host	Host details	Mean pairwise SNP
C1	P15	Plant	Spinach	0
	DI12_1	Animal	Turkey	
	DI8_1	Animal	Turkey	
	E10_1	Water	Water	
	H294_1	Human	Human	
	PG47_1	Animal	Poultry	
C2	P12	Plant	Cabbage	0
	BV23_1	Animal	Cattle	
	CH10_1	Animal	Dog	
	CH21_1	Animal	Dog	
	H242_1	Human	Human	
	H283_1	Human	Human	
	OI31_1	Animal	Goose	
	OI37_1	Animal	Goose	
	PC30_1	Animal	Pig	
	PC51_1	Animal	Pig	
	PC77_1	Animal	Pig	
	PG137_1	Animal	Poultry	
	PG366_1	Animal	Poultry	
	PP12_1	Animal	Poultry	
C3	P7	Plant	Salad	0
	CH89_1	Animal	Dog	
	OI47_1	Animal	Goose	
C4	P4	Plant	Salad	3
	E35_1	Water	Water	
	H7_1	Human	Human	

Table S3: Composition of ESBL-Ec transmission clusters (estimated with Phidelity) involving plant-isolated strains.
